# Repurposing methuosis-inducing anticancer drugs for anthelmintic therapy

**DOI:** 10.1371/journal.ppat.1012475

**Published:** 2024-09-05

**Authors:** Satish Kumar Rajasekharan, Vinothkannan Ravichandran, Bharath Reddy Boya, Anirudh Jayachandran, Jintae Lee

**Affiliations:** 1 Department of Biotechnology, School of Bioengineering, SRM Institute of Science and Technology, Chengalpattu District, Kattankulathur, Tamil Nadu, India; 2 Centre for Drug Discovery and Development (CD3), Amity Institute of Biotechnology, Amity University Maharashtra, Bhatan, Panvel, Mumbai, Maharashtra, India; 3 School of Chemical Engineering, Yeungnum University, Gyeongsan, Republic of Korea; University of Wisconsin Medical School, UNITED STATES OF AMERICA

## Abstract

Drug-resistant parasitic nematodes pose a grave threat to plants, animals, and humans. An innovative paradigm for treating parasitic nematodes is emphasized in this opinion. This approach relies on repurposing methuosis (a death characterized by accumulation of large vacuoles) inducing anticancer drugs as anthelmintics. We review drugs/chemicals that have shown to kill nematodes or cancerous cells by inducing multiple vacuoles that eventually coalesce and rupture. This perspective additionally offers a succinct summary on Structure–Activity Relationship (SAR) of methuosis-inducing small molecules. This strategy holds promise for the development of broad-spectrum anthelmintics, shedding light on shared molecular mechanisms between cancer and nematodes in response to these inducers, thereby potentially transforming both therapeutic domains.

## A battle against parasitic nematodes

A nematode’s dynamic adaptability and simple body structure make it remarkably resilient to harsh environmental conditions. Disease and death caused by parasitic nematodes in humans, livestock, and plants are enormous [**1**]. In recent years, pathogenic nematodes have evolved to adapt to many lifestyles and have shown remarkable ability to expand their host range [2]. Consequently, they are becoming more resilient to environmental conditions, host responses, and anthelmintics. Three decades after its discovery, ivermectin and its derivatives are still widely used to control and eradicate nematodes [3]. Ivermectin derivatives, for instance, function by preferentially paralyzing the nematodes, making them inert and unable to reproduce [4]. However, a few parasitic nematodes have already developed resistance to ivermectin, and resistance to these anthelmintic treatments is likely to emerge in the future [5]. Furthermore, it is possible for resistance genes to disseminate within clades. Hence, research ought to concentrate on screening anthelmintics that kill and destroy them or repurpose drugs that have cleared clinical trials. Here, we discuss a new therapeutic approach that involves repurposing anticancer drugs that could potentially kill nematodes via methuosis, a process of nematode and cell death marked by accumulation of vacuoles [6].

## Methuosis—A death by vacuolation

**Fig 1:**
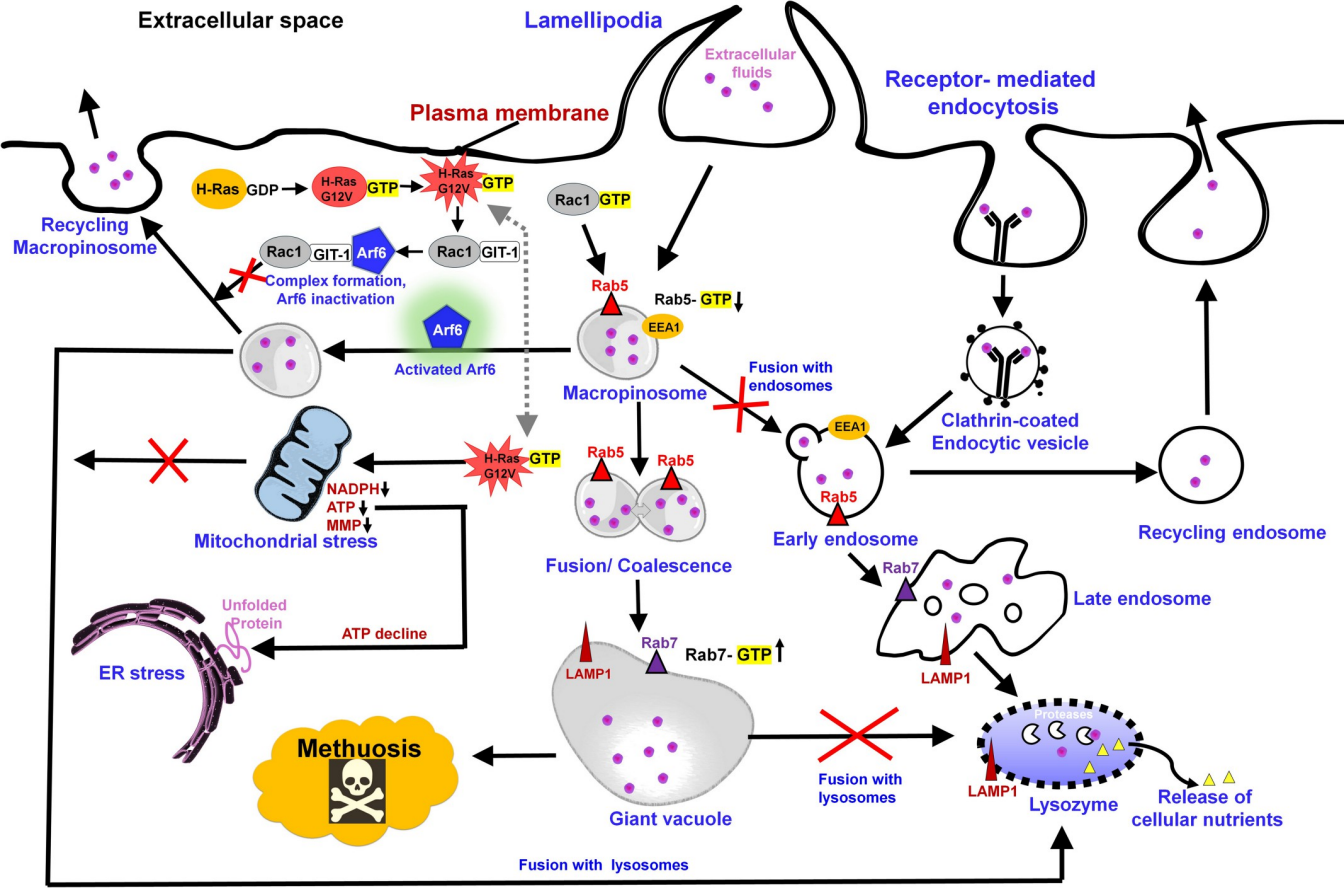
Molecular pathways that lead to methuosis in cancer cells. Briefly, Lamellipodia, or ruffles, allow nutrients and liquid tracer to enter cells, forming macropinocytic sinks, which coalesce into macropinosomes. The merger of macropinosomes produces giant vacuoles, which rupture and cause the death of cells by methuosis (refer text for details).

**Fig 2:**
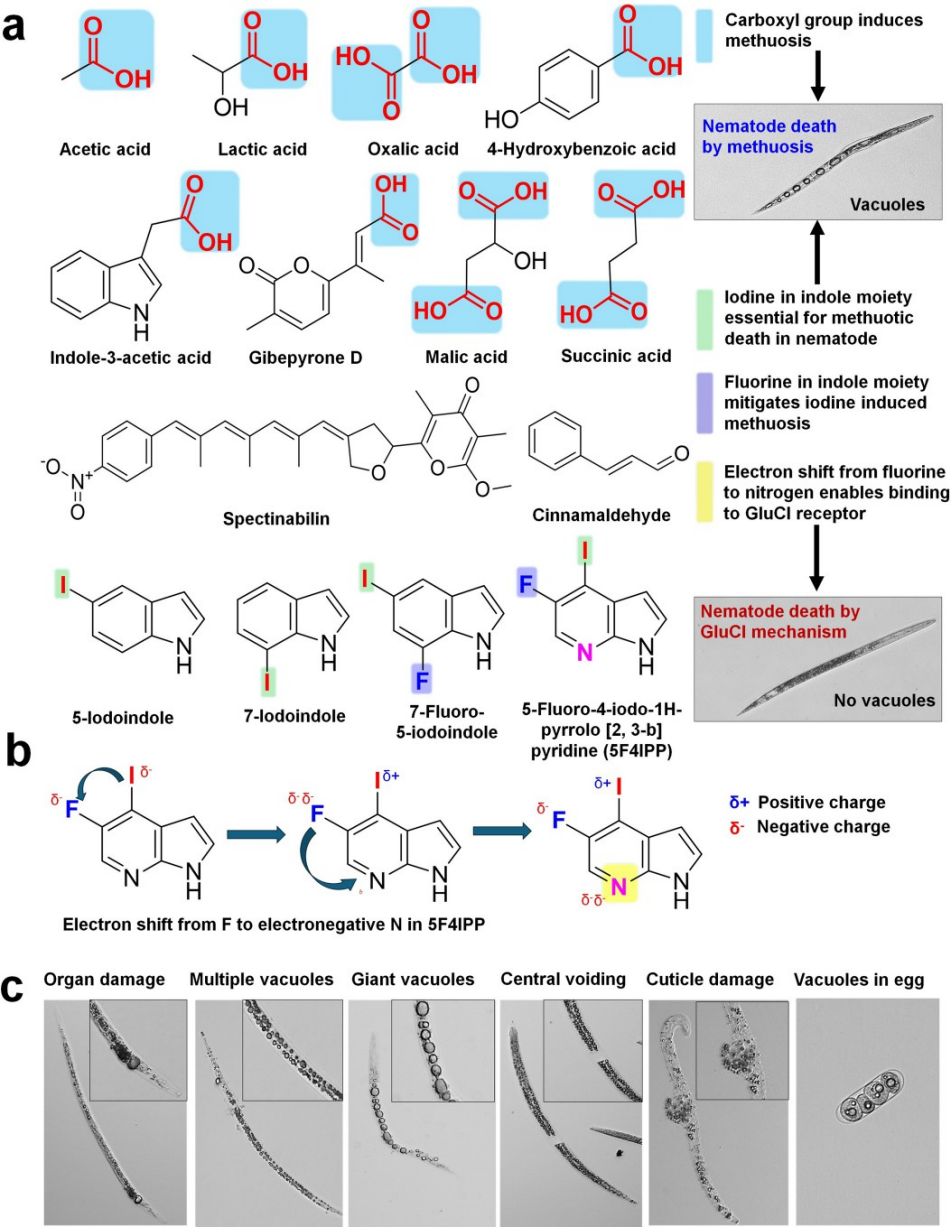
Organic acids with mono- or dicarboxy groups and indole derivatives with iodine or fluorine that cause vacuoles in nematodes (**a**), electronegative interactions between iodine and fluorine in 5F4IPP may be responsible for better glutamate-gated chloride channel (GluCl) receptor interactions and suppressed methuosis (**b**), and death phenotypes in pinewood nematode treated with 5-iodoindole (**c**).

Methuotic death in nematodes is characterized by formation of multiple tiny vacuoles, their subsequent fusion to form giant vacuoles, and the rupture of the cuticle layer [6]. Originally, methuosis was regarded as a nonapoptotic cell death phenotype derived from the Greek word “methuo” (to drink to intoxication) [7,8]. Methuosis and drugs that induce methuosis are extensively researched in cancer biology [9]. Multiple pathways have been reportedly associated with methuosis, with researchers actively engaged in bridging the existing knowledge gaps. The most studied pathways in cancer cells include the macropinosomes trafficking pathway governed by Ras cell signaling pathway [10]. The most striking characteristic of cells that undergo methuosis is the accumulation of large cytoplasmic vacuoles that are formed by the fusion of macropinosomes. Succinctly, following H-Ras overactivation, the cell develops a lamellipodia, or ruffle, which allows nutrients and fluid tracers to descend inside and form macropinocytic sinks. Further, macropinocytic sinks coalesce into macropinosomes through a cascade of GTPase activation. A typical scenario involves mature macropinosomes being recycled while some, expressing the late endosomal markers (Rab7 and LAMP1), fuse with endocytic pathway organelles such as endosomes and lysosomes, undergoing a sequential process of cell lysis and nutrient release [11]. During cancerous growth, macropinosomes fail to recruit early endosomal proteins, preventing them from fusing with lysosomes and recycling. Instead, they merge to form giant vacuoles that rupture and cause cell death by methuosis (Fig 1). In the last few years, several small molecules have been reported to induce methuosis in a variety of cancer cell lines (Table 1), while a few others were effectual in inducing vacuoles and methuotic death in nematode models (Fig 2). The text that follows will focus on these compounds that induce methuosis and provide a quick overview of the Structure–Activity Relationship (SAR) and mechanistic study with the aim to encourage repurposing anticancer drugs for anthelmintic therapy.

**Table 1:** Methuosis-inducing anticancer chemicals/agents that can be effectively repurposed for anthelmintic therapy.

Anticancer agents	Functional group (s)	Relevent function	Cancer cell lines	Death phenotype	Reference
Isobavachalcone	-Cl, -CO, -OH	V-ATPase, AKT	Myeloid cell lines (NB4, U937)	Methuosis-like cell death	[29]
Vacquinol-1	-Cl, -OH	Antitumor immune response	Human and rat glioblastoma models, RG2 and NS1	Macropinocytosis inducer	[21]
Tubeimoside 1	-OH, -CH_3_, -CO, -O-,-COO-	Inactivation of VEGF-A/VEGFR2/ERK signaling	SW480, CRC, NSCLC	Macropinocytosis hyperstimulation	[30]
Ursolic acid derivatives (C17)	-CN, -COOH, -CH_3_	Anticancer activity	HeLa cells	Macropinocytosis hyperstimulation	[18]
Indolyl-Pyridinyl-Propenone	-CH_3,_ -CO, -O-, -OH	PIKFYVE inhibitor	HCT116, U251 glioblastoma	Methuosis, microtubule disruption	[31]
Indole-based chalcones (MIPP, MOMIPP)	-CO, -CH_3_, -O-	Inhibition of endosomal trafficking, targeting Rab5 and Rab7	U251 glioblastoma, breast cancer cell	Methuosis	[32]
*Platycarya strobilacea* Sieb. Et Zucc (PSZ) (Extract)	n/a	Rac1 overexpression	Human nasopharyngeal carcinoma cells (CNE1 and CNE2 cells)	Methuosis	[33]
Jaspine B	-OH, -NH_2_, -C_14_	Ceramide synthase inhibitor	HGC-27 gastric cancer	Vacuolation related to methuosis	[34]
F14512	-CO, -NH_2_, -OH, -OCH_3_	Topoisomerase II inhibitor	A549 nonsmall cell lung cancer cells	Electron-lucent (methuosis-like)	[35]
DZ-514	-Br, -CO, -O-	Activation of ROS-MKK4-p38 axis	Breast cancer	Methuosis	[22]
Pyrimidinediamine derivatives (JH530)	-Br, -CO, -O-, -S-	Antitumor activitiy	Breast cancer	Methuosis	[36]
Tubeimoside-2	OH, -CH_3_, -O-, -COO-	MKK4-p38α Axis	Hepatocarcinoma cells	Methuosis	[37]
Spiropachysine A	-CO, CH_3_	Ras/Rac1 signal pathways	Hepatocellular carcinoma proliferation	Methuosis	[38]
Maduramicin	OH, -CH_3_, -OCH_3_, -O-,-COOH, NH_3_	Activation H-Ras-Rac1 signaling pathway	Myocardial cell H9c2	Methuosis	[39]
Silmitasertib (CX-4945)	-Cl, -COOH	Rac-1 activation	HepG2 cells	Methuosis	[40]
Epimedokoreanin C	-OH, -CO, -CH_3_	Regulation of Rac1 and Arf6	Lung cancer NCI-H292 and A549 cells	Methuosis-like cell death	[41]
Nutlin-3a	-Cl, -CO, -OCH_3_,—CH_3_	Inhibited the KRAS-PI3K/Akt-mTOR pathway	*KRAS* mutant NSCLC (nonsmall cell lung cancer) cells	Methuosis-like cell death	
L22	-NH_2_, -CH_3_	Interaction with PIKfyve kinase	HeLa and MDA-MB-231 cells	Methuosis	[36]
C13 (azaindole-based compounds)	-CO, -CF_3_, -CH_3_	-	MDA-MB-231, A375, HCT116, and MCF-7	Methuosis	[42]
DMBP (methyl 2,4-dihydroxy-3-(3-methyl-2-butenyl)-6-phenethylbenzoate)	-OH, -COO-, -CH_3_	Inhibited autophagic flux in cancer cells by inhibiting the function of VPS41	A549 and Panc-1 cell viability	Methuosis	[43]
Compounds **20** and **22**	-CO, -NH_2_	H-Ras activation	-	Methuosis	[44]
Microbial-derived amphiphilic CLP bacillomycin Lb (B-Lb)	-COOH, -OH, -CO, -NH_2_, -CH_3_	Triggered by cytoplasmic vacuolation through macropinocytosis	MDA-MB-231-Luc and MCF-7 cells	Methuosis-like cell death	[45]
2-Amino-14,16-dimethyloctadecan-3-ol	-OH, -NH_2_, -CH_3_	Disturbs later stages of endolysosomal process	HepG2	Vacuolation, partial macropinocytosis induction	[46]
HZX-02-059	-CF_3_, -CO, -CH_3_	PIKfyve and tubulin dual-target inhibitor	DHL cell lines WILL-2, LR, TMD8	Methuosis and cell cycle arrest	[47]
Ezetimibe	-F, -CO, -OH	NPC1L1 inhibitor	Human cancer cell line Du145/Du145TXR and MCF-7/MCF-7ADR cells	Methuosis	[48]
Glycosylated antitumor ether lipids (GAELs)	n/a	n/a	Epithelial cancer cell lines and BT474 cancer stem cells; MDA-MB-231, JIMT-1, and DU-145; MDA-MB-468, Hs578t, and MDA-MB-453 cell lines	Methuosis	[49]
Methanphetamine	-CH_3_	Ras and Rac1 activation	SH-SY5Y neuroblastoma cells	Hyperstimulation of macropinocytosis	[50]
BAPT compounds	-S	Endolysosomal trafficking defects that prevent recycling of lysosomes and cause lysosome-to-nucleus signaling defect	HCT-116 colon cancer cell line	Dual action Methuophagy	[51]
5-((4-(pyridin-3-yl)pyrimidin-2-yl)amino)-1H-Indole-2-Carbohydrazide derivatives (Compound **12A**)	-CO, -CH_3_	MAPK/JNK signalling pathway	HepG2, HeLa, MDA-MB-231, MCF-7, MCF-10A, LO2 cells	Methuosis	[52]
Bacoside A	-OH, -CH_3_, -O-	Excessive phosphorylation of calcium/calmodulin-dependent protein kinase IIA (CaMKIIA/CaMK2A) enzyme	GBM patient-derived glioblastoma cells	Hyperstimulation of macropinocytosis	[53]
Meridianin C	-Br, -NH_2_	Reducing the cellular level of Dickkopf-related protein-3 (DKK-3)	YD-10B human tongue cancer cells	Methuosis-like cell death	[54]
WJ-644A	Br^-^, -OCH_3_	Activation of unfolded protein response(UPR)	Human prostate cancer cell lines, DU145, PC3M, PC3, 22RV1, LNCAP, VCAP	Methuosis	[55]

## Vacuolar phenotypes and carboxyl functional groups

Vacuoles are the visual hallmark of methuosis in nematodes [6]. Vacuolar death was first spotted in plant parasitic nematode *Meloidogyne incognita* or the root-knot nematode, following treatment with carboxylic acids. Acetic acid, lactic acid, and their mixtures induced vacuolation in *M*. *incognita* juveniles [12]. Mixtures of organic acids consisting of acetic acid, lactic acid, malic acid, and succinic acid in *Lactobacillus brevis* WiKim0069 culture filtrates also induce vacuoles in *M*. *incognita* [13]. More pronounced phenotypes were observed when *M*. *incognita* J2 was treated with oxalic acid, a dicarboxylic acid [14]. Secondary metabolites from *Fusarium oxysporum* strain Fo162 that consisted of gibepyrone D, indole-3-acetic acid, and 4-hydroxybenzoic acid also induced vacuoles in *M*. *incognita* J2 [15]. Based on the SAR analysis, we speculate that the presence of carboxyl functional group as a key for the vacuolar phenotypes (Fig 2A). Carbonyl groups (C = O) and hydroxyl groups (O–H) make up the carboxyl group. The design and development of drugs relies heavily on compounds that contain carboxylic acids moieties [16]. Worldwide, more than 450 drugs with carboxylic acid moieties are marketed [17]. In most cases, carboxylic acid–containing drugs often trigger idiosyncratic reactions and cause idiopathic effects. There remains a lack of clear understanding regarding the mechanism of action. It is possible that vacuolation and methuosis contribute to disruption of cellular function, eventually causing death in nematodes. Two of the anticancer drugs containing carboxyl group, namely, ursolic acid (C17) and silmitasertib (CX-4945), induced methuosis in HeLa and colon cancer cell lines, respectively [18,19]. C17 specifically induced death by hyperstimulation of macropinocytosis, while CX-4945 triggered methuosis-like cell death accompanied by catastrophic vacuolation. Due to the presence of the carboxyl group, it is conceivable that both chemicals may induce similar vacuolation in nematodes, akin to their effect on cancer cells. In general, it would be interesting to repurpose small molecule inhibitors with carboxylic acid groups among the 450 FDA drug candidates for use as anthelmintics as well.

## Halogenated organic compounds and methuosis

The majority of anthelmintics contain one or more halogen substitutes [6]. We demonstrated that 5-iodoindole and 7-iodoindole selectively killed nematodes by triggering vacuolar phenotypes [6]. Iodine in the indole ring is the key factor in triggering methuosis, whereas fluorine (in 7-fluro 5-iodoindole) mitigates methuosis as an iodine antagonist (Fig 2B) [20]. Nematodes undergoing methuosis revealed several hallmarks and intriguing phenotypes. Small vacuoles formed inside the nematode’s body, which merged into larger ones and eventually ruptured, thereby killing the nematode. There was also evidence of cuticle damage, central voiding, and internal organ disruption in the nematodes and their eggs (Fig 2C).

Interestingly, many halogenated anticancer agents were found to have potential to induce methuosis and methuosis-like cell death (Table 1). It was first reported that a chalcone derivative named 3-(5-Methoxy-2-methyl-1H-indol-3-yl)-1-(4-pyridinyl)-2-propen-1-one (MIPP), along with its 5-brominated derivative (BMIPP), to trigger methuosis in glioblastoma cells [7]. They also found that MIPP possesses the ability to induce methuosis in various other cell lines, showing that these compounds have broad-spectrum activity. Vacquinol-1 (Vac), a quinolone derivative, was also reported to induce rapid methuosis-like cell death in glioblastoma cells [21]. The possible mode of action of Vac-induced methuosis is based on the ATP-inducible and carvacrol-sensitive ion channel TRPM7. Other compounds like meridianin A-G, an indole alkaloid, induced vacuolation by reducing the levels of Dickkopf-related protein-3 (DKK-3), a known negative regulator of macropinocytosis. CX-4945 (silmitasertib), a potent ATP-competitive inhibitor of CK2, with the unusual structural feature of having a free carboxylic acid and chlorine, could induce vacuolization in the cytoplasm of cholangiocarcinoma cells [19]. It is noteworthy to mention that CX-4945 was approved by the FDA for cholangiocarcinoma (bile duct cancer) in 2017 with an orphan drug designation [19]. DZ-514, a derivative of N-phenyl-4-pyrimidine diamine, induced time-dependent vacuolation in cancer cells, partially facilitated through the activation of the ROS-MKK4-p38 signaling pathway.

Exploring the potential of these small molecule inhibitors containing halogen groups that induce methuosis against nematodes as broad-spectrum nematicides would be intriguing. Our research, alongside studies on 5-iodoindole, Vacquinol-1, and DZ-514, respectively, indicates that these methuosis inducers have promising prospects for in vivo applications as well [22,23].

## Repurposing drugs: An unexplored panacea

Parasitologists, especially those in veterinary medicine, face a growing challenge of anthelmintic resistance [24]. Repurposing existing drugs as anthelmintics reduces the clinical trial burdens since drug screening is cumbersome, exorbitant, and time-consuming. The market offers a wide range of drugs that have passed clinical trials and are considered safe for use on plants, animals, and humans. Repurposing of an existing old drug/chemical offers possibilities of inexpensive, readily available solutions with extensive safety profiles. Although the repurposing approach is being pursued in many directions, we focalize on anticancer drugs that trigger vacuolation and cause methuosis-like death. While it would be challenging to establish a direct correlation in the mode of action of these drugs between nematodes and mammalian cells, there’s a flicker of hope that they could induce vacuolar death in nematodes. Furthermore, several recent studies suggest anthelmintic drugs may function as effective cancer therapeutics [25]. This is most likely owing to the fact that some helminths (intestinal parasitic helminths) can cause cancer and multiply rapidly in immunocompromised patients undergoing cancer chemotherapy [26–28]. The coexistence of cancer and helminth infections can be a circumstance necessitating drugs like methuosis inducers, which can mitigate both conditions. Currently, compounds like CX-4945 and MOMIPP are in various stages of clinical trials [19] but may be able to treat helminthic infections in the future. It may not be so farfetched to develop a panacea approach to these diseases. The repurposing of cancer drugs as anthelmintics and vice versa may be possible while simultaneously treating both conditions.

## Concluding remarks and future perspectives

In total, we discuss the biological effects and SAR analysis of small molecule methuosis inducers that may spur parasite death by causing methuosis. Methuosis-based therapeutic approaches have not been adopted against parasitic nematodes, so information on the topic is very limited. As we gain greater knowledge of the mechanisms of vacuolization in parasitic nematodes, we will be able to create more realistic perceptions of how parasites behave and respond to their environment. Repurposing strategies will encourage employing multiomics methodologies to explore the impact and mechanism of action of these methuosis-inducing anticancer agents against parasitic nematodes. Overall, this approach will likely pave the way for broad-spectrum anthelmintic and anticancer agents in the future as well as reveal the biological similarity between cancer cells and nematode cells in responding to these inducers.
